# INTERGENERATIONAL INTERACTIONS AND RELATIONSHIP QUALITY: A DAILY STUDY AMONG MOTHERS AND THEIR ADULT CHILDREN

**DOI:** 10.1093/geroni/igad104.3064

**Published:** 2023-12-21

**Authors:** Da Jiang, John Chi Kin Lee, Dexia Kong

**Affiliations:** Education University of Hong Kong, Hong Kong, Hong Kong; Education University of Hong Kong, Hong Kong, Hong Kong; The Chinese University of Hong Kong, Hong Kong, Hong Kong

## Abstract

The parent–child relationship is one of the longest-lasting and closest relationships in human life. Because of its long-lasting nature, more studies focus on the long-term exchanges between parents and children. Few studies have examined the effects of daily interactions on relationship quality. However, such an understanding is crucial during public health emergencies, when daily support amongst close family members is critical for life quality and even survival. Using data collected during the COVID-19 pandemic, we examined the relationship between daily support and relationship quality among middle-aged and older mothers and their adult children. Seventy-seven dyads of mothers (age range: 44–80 years, Mage = 53.78, SDage = 9.57) and adult children (age range: 18–54 years, Mage = 26.61, SDage = 9.46) reported their daily exchanges with their child/parent (i.e., the support they had received from and provided to the other) and daily relationship quality (i.e., relationship satisfaction and trust) each day for 14 consecutive days. Receiving support was positively associated with relationship satisfaction and trust on both Day N and Day N+1 in mothers, and it was positively associated with relationship satisfaction on Day N and trust on both Day N and Day N+1 in children. Offering support was positively associated with relationship satisfaction on Day N (but not Day N+1) for both mothers and children. Offering support was not associated with daily trust in either mothers or children. The findings highlight the importance of daily support on relationship quality during a public health emergency.

